# Incorporating Negative Sample Training for Ship Detection Based on Deep Learning

**DOI:** 10.3390/s19030684

**Published:** 2019-02-07

**Authors:** Lianru Gao, Yiqun He, Xu Sun, Xiuping Jia, Bing Zhang

**Affiliations:** 1 Key Laboratory of Digital Earth Science, Institute of Remote Sensing and Digital Earth, Chinese Academy of Sciences, Beijing 100094, China; gaolr@radi.ac.cn (L.G.); heyq@radi.ac.cn (Y.H.); zb@radi.ac.cn (B.Z.); 2 College of Resources and Environment, University of Chinese Academy of Sciences, Beijing 100049, China; 3 CETC Key Laboratory of Aerospace Information Applications, Shijiazhuang 050081, China; 4 School of Engineering and Information Technology, The University of New South Wales, Canberra Campus, Canberra, ACT 2006, Australia; x.jia@adfa.edu.au

**Keywords:** ship detection, deep learning, negative sample training, sea–land segmentation, high-resolution satellite images

## Abstract

While ship detection using high-resolution optical satellite images plays an important role in various civilian fields—including maritime traffic survey and maritime rescue—it is a difficult task due to influences of the complex background, especially when ships are near to land. In current literatures, land masking is generally required before ship detection to avoid many false alarms on land. However, sea–land segmentation not only has the risk of segmentation errors, but also requires expertise to adjust parameters. In this study, Faster Region-based Convolutional Neural Network (Faster R-CNN) is applied to detect ships without the need for land masking. We propose an effective training strategy for the Faster R-CNN by incorporating a large number of images containing only terrestrial regions as negative samples without any manual marking, which is different from the selection of negative samples by targeted way in other detection methods. The experiments using Gaofen-1 satellite (GF-1), Gaofen-2 satellite (GF-2), and Jilin-1 satellite (JL-1) images as testing datasets under different ship detection conditions were carried out to evaluate the effectiveness of the proposed strategy in the avoidance of false alarms on land. The results show that the method incorporating negative sample training can largely reduce false alarms in terrestrial areas, and is superior in detection performance, algorithm complexity, and time consumption. Compared with the method based on sea–land segmentation, the proposed method achieves the absolute increment of 70% of the F1-measure, when the image contains large land area such as the GF-1 image, and achieves the absolute increment of 42.5% for images with complex harbors and many coastal ships, such as the JL-1 images.

## 1. Introduction

Ship detection plays a critical role in maritime management, dynamic surveillance of harbors, ship rescue, etc. Remote sensing can efficiently acquire images covering the vast area of ocean, which provides unique advantages for ship detection at a large-scale. With the development of the aerospace industry, various countries have rushed to launch high-resolution imaging satellites, and the capacity of detection equipment mounted on them has also rapidly increased. This means that a large amount of raw data and good development prospect for ship detection. Due to their large-scale and high-resolution; however, we must face the challenge of handling more complex and massive data, including complicated background.

Kanjira et al. [1] provided a review based on 119 ship detection and classification papers from 1978 to 2017. From the review we can see that there are two main types of methods for traditional ship detection. One type of method is to extract the preset features such as the shape [2,3,4], aspect ratio [5], or area of the connected region after pretreatments including sea–land segmentation. In order not to lose the details of the object, another type of method is to extract features directly using all pixels in the image [6]. These two types of features are then used as basic information for ship detection. In these methods, the performance of ship detection depends on the design of the features. However, the method of artificially designing specific features is not robust enough to extract effective features constantly, as well as requiring a very rich professional knowledge, a large amount of manpower, and material resources. Meanwhile, the method of extracting features using all pixels is too cumbersome and brings in a large amount of redundant information. Therefore, artificial feature design is increasingly difficult to adapt to the current situation in which the amount of information is soaring.

With the development of computer vision, deep learning has achieved the great success in a wide range of problems in the past few years, such as object detection [7], classification [8,9], and semantic labeling [10]. In the field of remote sensing, many deep learning-based methods have also been proposed for object detection [11,12,13,14,15,16,17], such as oil tank [12,13], airplane [14,15], and vehicle [16,17]. A great breakthrough has been made in ship detection based on deep learning. Meanwhile, massive high-resolution images have many detailed texture information, which coincides with the fact that deep learning requires a large number of training samples and detects objects based on image texture information. Therefore, it is suitable to adopt the deep learning method to solve the ship detection problem using large-scale high-resolution optical images.

Deep learning-based ship detection in high-resolution optical images has attracted the attention of researchers in recent years and achieved a significant improvement. Zhang et al. designed a shallow neural network for ship detection based on a convolutional neural network (CNN) called S-CNN, which combines CNN with the ship head model and the ship body model [18]. Some researchers also designed new methods based on the combination of multiscale rotation region detection and deep learning [19,20], which had a good effect in the task of detecting densely arranged ships, but it also caused more false alarms. The current target detection method based on deep neural networks can be roughly divided into two types. One is the method of region proposal methods [21,22] represented by Faster R-CNN [21], which generates a set of potential bounding boxes then run classifiers to determine whether they contain targets. The other one reframes object detection as a regression problem and predicts coordinates of bounding boxes and class probabilities directly from the image features, such as You Only Look Once (YOLO) [7] and Single Shot MultiBox Detector (SSD) [23]. The major advantage of the second type of method is fast detection, yet the limitations are also obvious in which it may output incorrect localizations and it is hard to detect small targets. Unlike the general objects in the natural scene images, the size of ships in the remote sensing images is relatively small. Given that Faster R-CNN is more suitable for small target detection, the study of the detection of ships presented in this paper is based on Faster R-CNN. Inspired by Faster R-CNN, Yao et al. proposed a method which used CNN and region proposal network (RPN), in which the anchors were designed by intrinsic shape of ship targets [24]. However, this method only analyzes the situation of ships on the sea, and does not consider the complexities of land or harbors.

The deep learning-based methods mentioned above were applied to small images of the harbors or the sea surface. The majority of traditional ship detection methods were developed using a small number of images, and mostly the images taken in a calm sea state [1]. Here, it should be noted that two problems in ship detection for large-scale high-resolution remote sensing images are remained: the false alarm caused by a large area of nearby land and heterogeneous background of the land [1]. As some land areas share similar intensity and texture distributions to ships, there will be a large number of false alarms when we detect ships from large-scale remote sensing images [25].

Current ship detection methods usually conduct sea–land segmentation before extracting ship features [25,26,27,28,29] to solve these problems. Following the segmentation, we can use deep network to extract features of ships. Sea–land segmentation generally has two solutions: using available GIS layers of coastline and using grayscale and texture information of the images [29]. However, these two methods have some disadvantages, respectively. When GIS layers is used for segmentation, they may not be up to date, as the layout of port is constantly adjusted, and the coastline changes with the seasons. With the improvement of the resolution of optical remote sensing images, the limited spatial resolution of the existing geo-location information database can no longer meet the demand for fine sea–land segmentation. Segmentation based on grayscale and texture [30,31,32] is not only sensitive to the parameters selected, but also prone to misclassification. Also, segmentation using grayscale and texture information needs to be performed on the entire image. When we process an entire image, a large amount of calculate resources and time will be sacrificed. In recent years, the method of sea–land segmentation based on deep learning also emerges. It can avoid handcrafted extraction of features, but the detection of the parked ship and the large ship is still challenging [33]. Moreover, the preprocessing steps of sea–land segmentation increase the complexity of the algorithm.

As deep learning is suitable for a large number of images to extract features, it provides high generality and capacity in target detection [9,19]. Deep learning network uses nontarget objects as negative samples to learn their texture features. Thus, when nontarget objects of the same category are subsequently detected, the network is trained to recognize them as background area. Therefore, another way to remove false alarms is to use the nonship objects that look like ship as negative samples to train the network. In the previous methods, docks, small islands, and other ship-shaped coastal buildings were used as negative samples to extract their features to remove false alarms [4,5,28]. To improve the performance of Faster R-CNN, Li et al. proposed hard negatives, which were prone to be detected falsely by the detector [22]. However, these methods need to specifically select and label the sensitive negative samples.

To solve the problems mentioned above, this paper proposes a ship detection method which incorporates negative sample training based on Faster R-CNN for large-scale optical images without sea–land segmentation. To avoid the false alarm on land, it adds a large number of random negative sample images (patches) containing only land area to train the network. It is different from the selection of negative samples by targeted way in other deep learning-based ship detection methods, such as the method proposed by Jianwei Li et al. [22]. Due to the strong ability of deep learning to extract features from massive images and convert them into useful information, the false alarm caused by the land area in the large images can be avoided without the preprocessing of sea–land segmentation. The proposed method of incorporating negative sample training makes the avoidance of false alarms not limited to certain types of objects that are more prone to produce false alarms, but all terrestrial objects. 

The reminder of this paper is organized as follows. In Section 2, two detection methods based on deep learning and two sea–land segmentation methods used in this paper for comparison are detailed. In Section 3, the processing steps of ship detection based on Faster R-CNN incorporating negative sample training proposed in this paper are presented in detail. Section 4 presents the data and experiment settings and experimental results. Section 5 discusses the effectiveness of the proposed negative sample training method based on deep learning with different backgrounds for detection, and its quantitative comparison with sea–land segmentation. Section 6 draws the conclusions.

## 2. Related Works

### 2.1. Related Detection Networks

In this section, we present a brief introduction of Single Shot MultiBox Detector (SSD) [23] and FPN [34] which are deep learning-based detection methods for comparative experiments.

#### 2.1.1. SSD: Single Shot MultiBox Detector

SSD is a method for detecting objects in images using a single deep neural network, which discretizes the output space of bounding boxes into a set of default boxes over different aspect ratios and scales per feature map location. At prediction time, the network generates scores for the presence of each object category in each default box and produces adjustments to the box to better match the object shape.

SSD is simple relative to methods that require object proposals because it completely encapsulates all computation in a single network. It makes SSD easy to train and fast to detect, and straightforward to be integrated into systems that require a detection component. However, it has low performance in detecting small objects, such as ship targets, although it has a fast detection speed.

#### 2.1.2. FPN: Feature Pyramid Networks

FPN is a topdown architecture with lateral connections developed for building high-level semantic feature maps at all scales, which shows improvement as a generic feature extractor in several natural image applications based on Faster R-CNN system [19].

FPN uses feature pyramid representations for multiscale problem in natural images, and combines the high-resolution shallow-layer features and the high-semantic deep-layer features. It provides a practical solution for research and applications of feature pyramids, without the need of computing image pyramids.

### 2.2. Sea–Land Segmentation

Due to the change of coastline, it does not mean that every target scene of remote sensing image must have corresponding GIS data that is close to its acquisition time. It clearly limits the use of methods based on GIS data. Also, segmentation based on grayscale and texture is not only sensitive to the parameters selected, but also consumes a large amount of calculate resources and time. Therefore, this section introduces a segmentation method based on the deep learning network, fully convolutional network (FCN) [33].

**Figure 1:**
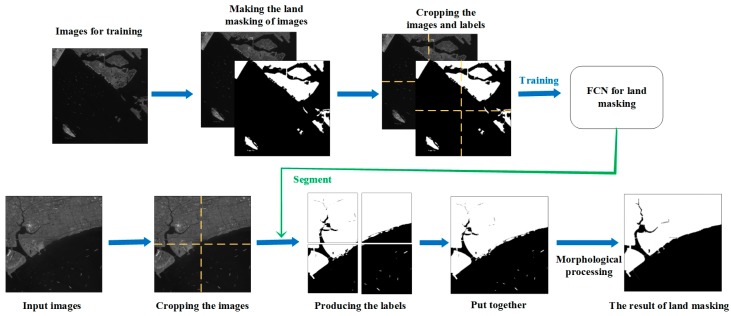
The process of land masking based on the fully convolutional network (FCN).

We use the FCN convolutional neural network as proposed previously [33] and implement it with TensorFlow. Figure 1 subtly depicts the process of using FCN for segmentation. This method is divided into four steps.

Firstly, training set collection. Some optical images of similar resolution are required to adjust the model by making sea–land segmentation labels of images manually.

Secondly, is FCN network training. The training set of images and corresponding labels are cut into chips (1000 × 1000 in this paper), which feed a fully convolutional network to generate a new model for sea–land segmentation.

Thirdly, land masking using trained model. The images to be detected are divided into small chips which have the same size as training set. Then, the trained network takes these image chips as input and produces their labels. Eventually, the cropped labels are put together to obtain the preliminary sea–land segmentation map.

Fourthly, morphological processing. For an area that is determined to be land by preliminary segmentation, if the area is smaller than the threshold we set and surrounded by ocean areas, it will be redetermined as a sea area. In the same way, the areas determined to be oceans which are smaller than the threshold are redetermined as land area. Refer to the size of the ship on the image, we optimize the threshold parameters by multiple times. Finally, the segmented image is mapped into the original image, and the land area is replaced with a value of 0. The ocean area remains unchanged. Thus, the sea–land segmentation is finished.

## 3. Proposed Method

**Figure 2:**
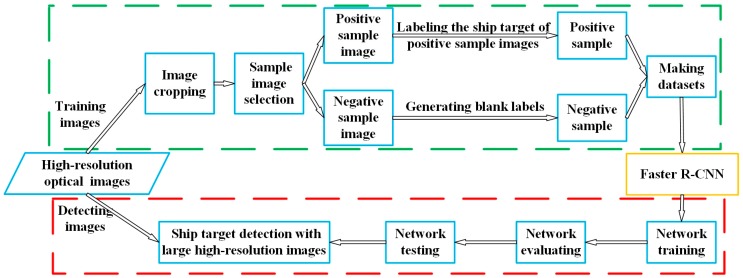
Faster R-CNN-based flow chart of our algorithm.

The overall processing steps of the deep learning-based ship detection method proposed in this paper are shown in Figure 2. This section will give a detailed introduction to the Faster R-CNN-based ship detection network (yellow box) and the reason of effectiveness by using negative sample images to reduce false alarms in Section 3.1, negative sample training (green dotted box) in Section 3.2, and network training and detection (red dotted box) in Section 3.3.

### 3.1. The Framework of Faster R-CNN-Based Ship Detection

**Figure 3:**
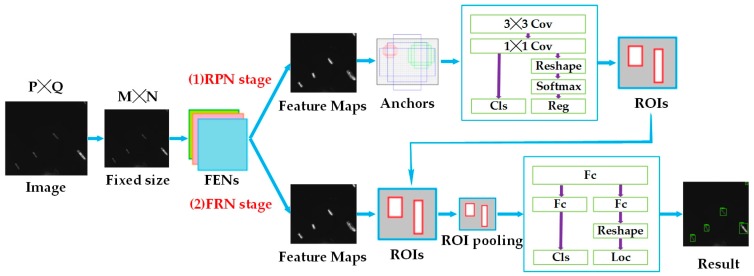
The network structure of the Faster R-CNN-based ship detection method.

In terms of structure, Faster R-CNN incorporates feature extraction, region proposal, bounding box regression, and classification into a single network (shown in Figure 3), which greatly improves the overall performance of the network. There are two main routes: region proposal network (RPN) stage and fast R-CNN (FRN) stage. RPN stage provides RoIs for FRN stage, and they share the feature extraction networks (FENs).

From the training image, we assign a binary class label of being a ship or not to each anchor for training RPNs, and assign a positive label to two kinds of anchors: (1) the box(es) with the highest Intersection over Union (IoU) with any ground-truth box and (2) the box(es) with an Intersection over Union (IoU) overlap higher than 0.7 with any ground-truth box. Moreover, a negative label is assigned to a nonpositive anchor if its IoU ratio is lower than 0.3 for all ground-truth boxes. With these definitions, an objective function following the multitask loss in Fast R-CNN [21] is requested to be minimized. The loss function in this paper is defined as

(1) $$L(\{p_{i}\},\{t_{i}\})=\frac{1}{N_{cls}}\sum_{i}L_{cls}(p_{i},p_{i}{}^{*})+\lambda \frac{1}{N_{reg}}\sum_{i}p_{i}{}^{*}L_{reg}(t_{i},t_{i}{}^{*}),$$

where, i is the index of an anchor in a minibatch and $p_{i}$ is the predicted probability of anchor i being an object. The ground-truth label $p_{i}{}^{*}=1$ if the anchor is positive and $p_{i}{}^{*}=0$ if the anchor is negative. $t_{i}$ is a vector representing the 4 parameterized coordinates of the predicted bounding box, and $t_{i}{}^{*}$ is that of the ground-truth box associated with a positive anchor.

(2) $$L_{cls}(p_{i},p_{i}{}^{*})=-\mathrm{lg}[p_{i}p_{i}{}^{*}+(1-P_{i}{}^{*})(1-P_{i})].$$

(3) $$L_{reg}(t_{i},t_{i}{}^{*})=R(t_{i}-t_{i}{}^{*}).$$

(4) $$smooth_{L1}(x)=\{\begin{array}{ll} 0.5x^{2} & |x|<1\\ |x|-0.5 & |x|\geq 1 \end{array}.$$

The classification loss $L_{cls}$ is log loss over two classes (ship vs. not ship), and can be calculated through Equation (2) [21]. For the regression loss $L_{reg}$, which is shown in Equation (3) [21], R is the robust loss function (smooth $L_{1}$) in Equation (4) [21]. The two terms are normalized by $N_{cls}$ and $N_{reg}$, and weighted by a balancing parameter λ.

When the network trains a land-based anchor which is similar to a ship, the ground-truth label $P_{i}{}^{*}=0$. According to Equation (2), it can be known that $L_{cls}(p_{i},p_{i}{}^{*})=-\mathrm{lg}(1-P_{i})$. So that $L_{cls}$, which means the value of the classification loss generated by this anchor, is larger as the larger of $p_{i}$. Therefore, it can improve the performance of detecting ship analogs on land, so as to leave out the step of land–sea segmentation.

### 3.2. Negative Sample Training for Faster R-CNN-Based Ship Detection

Based on the characteristics that deep learning is suitable for a large number of images to extract features, a large number of images not only proves the generality and significance of methods, but is also an important factor to increase the detection performance. As described in Section 3.1, the proposed network based on Faster R-CNN uses images of land areas as negative samples to learn their texture features. Thus, when land areas are subsequently detected, the network is trained to recognize them as nonship background area.

**Figure 4:**
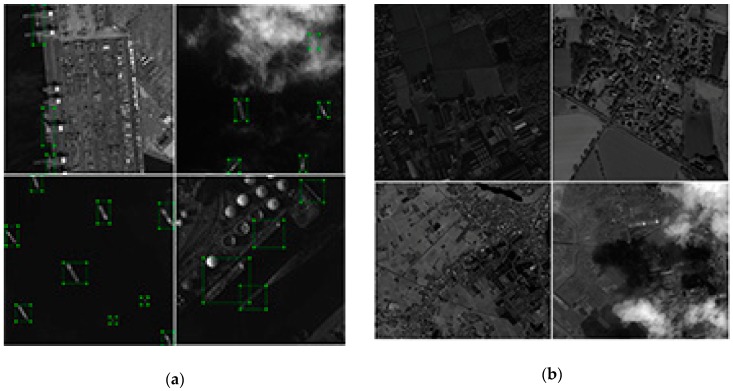
Positive sample images and negative sample images used in network training. (**a**) Positive sample images. (**b**) Negative sample images.

Therefore, we input a large number of random negative sample images containing only land area without any ship targets to train the network to avoid the false alarm on land without the segmentation of land and sea. In order to reduce the job of labeling negative samples, as shown in Figure 4, our negative sample image does not contain any ship targets, and the entire image is used as a nonship target to train the network. When we label ship targets, the negative sample images are separated from the positive sample images. So that the negative sample images only need to generate blank labels.

The flow path of the negative sample training (shown in the green dotted box in Figure 2) can be divided into the following steps.

(1) Select images. The images used to make positive and negative sample images adopts high spatial resolution data with resolutions of the level of meters or submeters, and are consistent with the band of the images to be detected after training the network.
(2) Crop images and pick positive and negative sample images. Crop the images to T×T-size images. From these images, select N images containing ship targets (as shown in Figure 4a) to form a positive-sample-image set $U={\left\{U_{i}\right\}}_{i=1}^{N}$, and M images that do not contain any ship targets (as shown in Figure 4b) to form a negative-sample-image set $V={\left\{V_{i}\right\}}_{i=1}^{M}$.
(3) Generate labels of positive and negative sample images. Mark the position of ship target in positive sample images and generate a set of labels $W={\left\{W_{i}\right\}}_{i=1}^{N}$ corresponding to the image set U, where $W_{i}={\left\{\left(x_{1t},x_{2t},y_{1t},y_{2t}\right)\right\}}_{t=1}^{N_{i}}$ denotes the set of ship positions in the i-th image of set U, and $N_{i}$ denotes the number of ships in the i-th image of set U. $\left(x_{1t},x_{2t},y_{1t},y_{2t}\right)$ represents the coordinate position of the t-th ship in an image. The negative-sample-image set V has no ship target, so it is not necessary to annotate it; blank labels corresponding to the negative sample images can be generated directly.
(4) Make datasets. The sets U, V, and W are randomly divided into $\left\{U^{\left(1\right)},U^{\left(2\right)},U^{\left(3\right)}\right\}$, $\left\{V^{\left(1\right)},V^{\left(2\right)},V^{\left(3\right)}\right\}$, and $\left\{W^{\left(1\right)},W^{\left(2\right)},W^{\left(3\right)}\right\}$, respectively, according to a certain proportion. The set $\left\{U^{\left(1\right)},V^{\left(1\right)},W^{\left(1\right)}\right\}$ represents the training dataset, the set $\left\{U^{\left(2\right)},V^{\left(2\right)},W^{\left(2\right)}\right\}$ represents the evaluating dataset. Since the network test step is a simulation for subsequent ship detection of large images, and the assessment of the test result need to be manually identified and calculated, the test data set only contains the image set $\left\{U^{\left(3\right)},V^{\left(3\right)}\right\}$.
(5) Network training. Using the dataset $\left\{U^{\left(1\right)},V^{\left(1\right)},W^{\left(1\right)}\right\}$ to train Faster R-CNN-based network built according to Section 3.1.

### 3.3. Network Training and Detection

**Figure 5:**
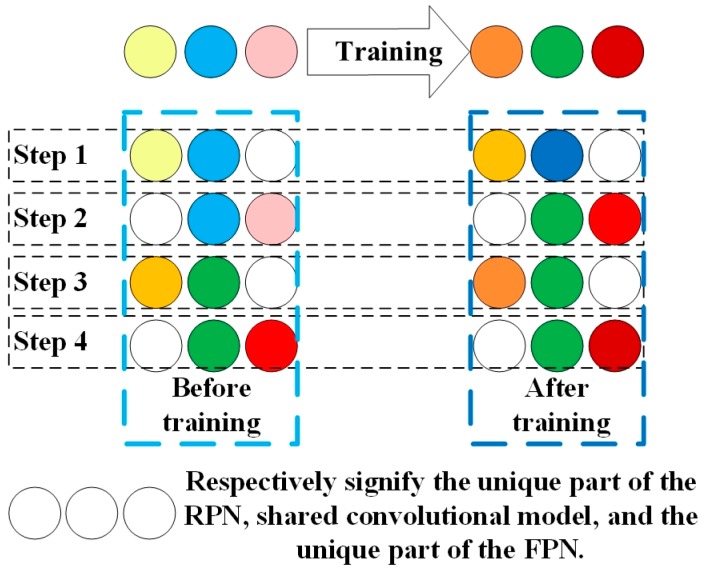
The method of alternating training regional proposal network (RPN) stage and fast R-CNN (FRN) stage.

Use dataset to train the RPN part and FRN part for the Faster R-CNN-based network was built according to Figure 3. This network is based on the stochastic gradient descent principle, and adopts the method to learn shared features via alternating training RPN stage and FRN stage as shown in Figure 5. The whole process is divided into four steps. The left side of the figure shows the network before each step of training, and the right side shows the network after each step of training. The entire network is represented by three small balls of different colors. The same color indicates that this part of the network has the same parameters.

As seen from Figure 5, the top three balls on the left represent the network that is initialized by migration learning before training. The top three balls on the right indicate the network after four steps of training. The following four lines describe the four-step training in detail. Specifically, in step 1, ImageNet pretraining model is used to initialize the RPN network parameters (including ball 1 and ball 2), then the RPN is trained by using the back propagation algorithm and optimized. After training, the convolutional model shared by RPN and FRN (ball 2), as well as the unique part of the RPN (ball 1), will be updated (changed color). The training of step 2 is similar to step 1 in training the FPN stage. In step 3, as it can be seen from the figure, in the network before training, the ball 1 is from step 1 and the ball 2 is from step 2, which means the shared convolutional model from step 2 and the unique part of the RPN from step1 are used to initialize. Then, we maintain the parameters of the convolution layer unchanged, and fine-tune the remaining part of the RPN. During this training process, the shared convolutional model remains unchanged and is therefore called sharing, while the unique part of the RPN is changed. The network training in step 4 is similar to step 3.

After the network is trained and the parameters are adjusted to the best, the test part of the dataset is used for testing. We input the image into the network, and output the target result to the image when a score greater than a certain threshold score, where 0.5 is defined in this paper.

Finally, when detecting the ship in the image, the large-scale high-resolution images are input into the trained network block by block according to a certain size (preferably close to the size of the images used to train the network). In order to prevent the missed detection of the ships which across the blocks, we leave an overlap of a certain pixel width between blocks. The images are input into the network for ship detection, and we get the position and confidence of each ship. Ultimately, our measurement of the performance of its detection also depends on recall and precision of the results.

## 4. Results

In this part, we present the experimental results of our paper in three parts. In Section 4.1, we present the results of comparative experiments of three deep learning-based detection networks. In Section 4.2, we combined Faster R-CNN, which shows the best performance in the Section 4.1, with the negative sample training proposed in this paper to achieve ship detection for large-scale images without the sea–land segmentation. We also show comparative experimental results of the proposed method with the FCN sea–land segmentation-based method. Section 4.3 shows a detailed analysis of different test results for different images.

We used i5-7500 CPU, 16 GB RAM hardware, 6 GB Graphics card of NVIDIA 1060 and the TensorFlow framework under Linux to carry out these experiments.

### 4.1. Comparative Experiments of Detection Networks

To the best of our knowledge, there is no public ship dataset available for the methods test. To facilitate the research, We collected and cut some Gaofen-1 (GF-1) and Gaofen-2 (GF-2) satellite images to small size and then built them into a dataset including training, evaluating, and test datasets (as shown in Table 1). Figure 4 shows some images as an example.

The detection results of SSD, FPN, and Faster R-CNN on our dataset described above are shown in Table 2. The migration learning method is used on SSD to train sufficient on our dataset.

Since FPN and Faster R-CNN have similar structures, Res101 network are used in both, and other training parameters are also the same, for example, batch size (the number of image processed each time) is 2, learning rate (determine the convergence effect of the model) is 3, and max epochs (the round numbers of calculate, the larger the value, the easier it is to converge) is 12.

The indicator of the evaluation performance is mAP (the mean average precision) [21]. It can be seen from the comparison results that although the Faster R-CNN method has a slower detection speed, the detection performance is the best. Therefore, we choose Faster R-CNN as the basic network to incorporate negative sample training for ship detection in this paper.

### 4.2. Results of Negative Sample Training for Faster R-CNN-Based Ship Detection

In order to analyze the influence of different proportions of positive and negative samples on the detection performance, we used Faster R-CNN, which attained the best detection performance in the previous comparison, combined with the negative sample training to train four networks with different proportions of positive and negative samples (as shown in Table 3).

Sample images which are shown in the previous section including positive sample images and a certain amount of negative sample images (as shown in Figure 4b) are used to train the networks. The four networks are trained until the parameters are close to optimal, that is, the network loss values are not significantly reduced. The training parameters are introduced in Table 3.

**Figure 6:**
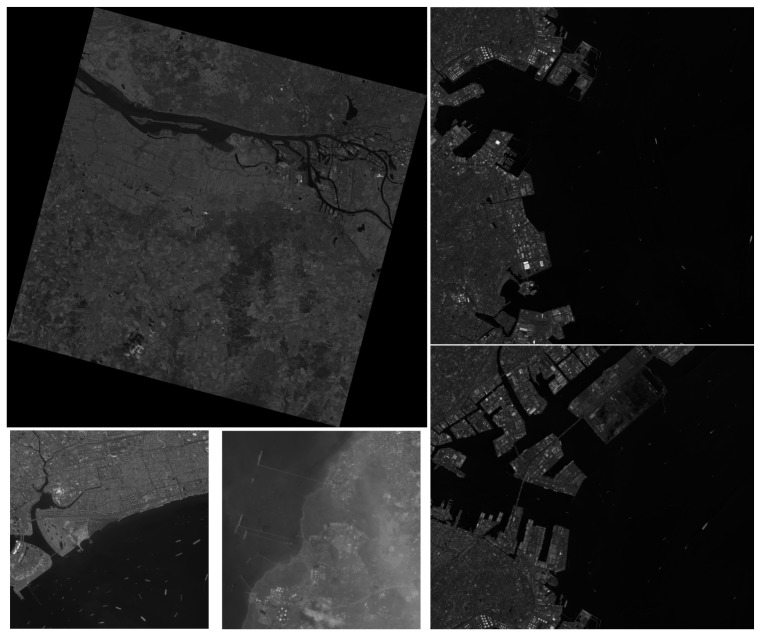
The five test large images. The images on the left are GF-1, GF-2(1), and GF-2(2), and the images on the right are JL-1(1) and JL-1(2).

In order to test whether incorporating the negative samples training method proposed in this paper can reduce the false alarm on land for large images, we collected five large images taken by GF-1, GF-2, and Jilin-1 (JL-1) satellites. Table 4 and Figure 6 show the information of images.

As shown in Figure 6, the test images we used include GF-2 images sheltered by thin clouds with the same proportion of the land and sea area, GF-1 images with a large proportion of land areas, and JL-1 images where the sea area accounts for a large area and the layout of harbors are more complex.

**Figure 7:**
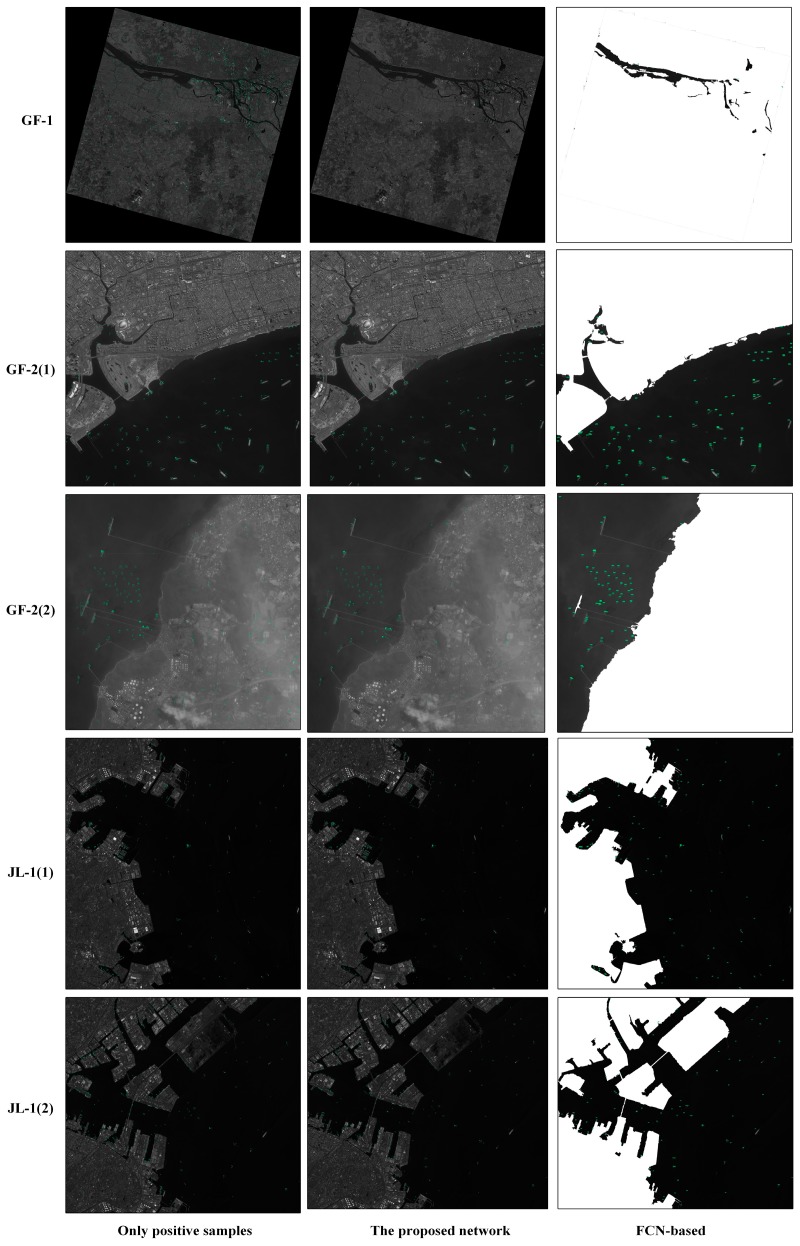
Images of partial experimental results. The second column represent the results of ship detection using networks trained by positive and negative sample images with the proportion of 1:1.

Afterwards, using the trained networks, we design experiments to compare the method of negative sample training with a method that uses the same networks to detect ships after preprocessing of sea–land segmentations of five large images with different resolutions, different sizes, and different background, including one GF-1 image, two GF-2 images, and two JL-1 images (Figure 7 shows the experimental results). 

**Figure 8:**
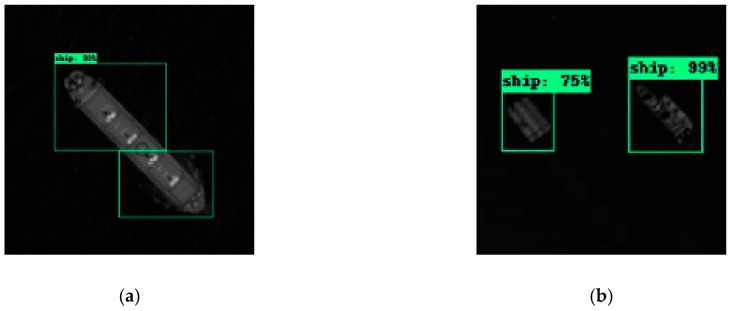
The diagram of judgement used to calculate the test result. (**a**) Schematic diagram of the same ship being detected twice. (**b**) Schematic diagram of multiple parallel ships being detected only once. We considered that only one ship was detected if these conditions occur.

We need to identify the image block-by-block when detecting ships in a large-scale image. Thus, in order to prevent ships from being missed just on the boundary between blocks, we set a 200-pixel overlap between each block. However, this also aggravates the situation that the same ship is repeatedly detected, as shown in Figure 8a. In this case, when we calculate the recall and precision, we considered that only one ship was detected. If there is only one exterior matrix for the parallel ships in the test results as shown in Figure 8b, we also think that the network only detected one ship.

### 4.3. Comparison by Using Different Images

In our experiments, the commonly used indicators are calculated for measuring the performance of ship detection: recall (R), precision (P), and F1-measure (F) [19]. At the same time, we recorded the time required for the experiment. Table 5, Table 6, Table 7, Table 8 and Table 9 show the detection performance and the time consumption of detections. From Figure 7, we can see that there are some differences in the detection results of the five images. We will introduce them separately.

(1) Panchromatic GF-1 image with a spatial resolution of 2 m and size of 21,227 × 21,227.

**Figure 9:**
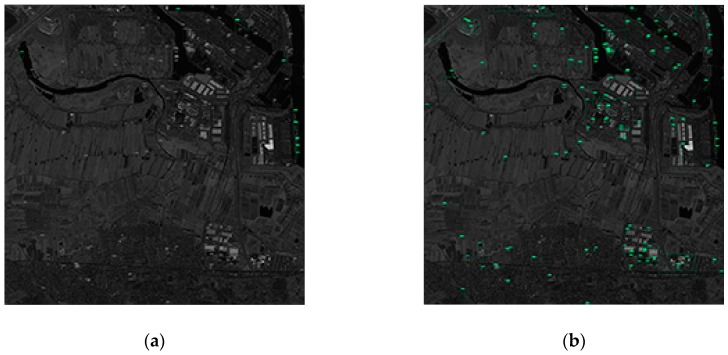
The detection results of the partial region of the GF-1 image. (**a**) Test results using network with a proportion of positive to negative samples of 1:1. (**b**) Test results using network with a proportion of positive to negative samples of 1:0.

Since the spatial resolution of the GF-1 image is large and the water area contained in the image is only rivers, where ships in river are relatively small, the detection performance of the image is worse (as shown in Table 5). This image contains a large proportion of land areas, so that the method incorporating negative sample training avoids most false alarms from the land. Therefore, a Faster R-CNN-based ship detection network with negative sample training has been greatly improved compared to network which is trained without negative samples; and due to the large proportion of land area, it can be seen that networks which add more negative samples have better detection performance. As the large area of the image, a large amount of time is consumed in the sea–land segmentation process, and the detection performance of the sea–land segmentation method are not very good due to the poor separation effect between the river and the land. Figure 9 shows the detection results of the partial region of the GF-1 image.

(2) The first panchromatic GF-2 image with a spatial resolution of 0.81 m and size of 10,000 × 10,000.

**Figure 10:**
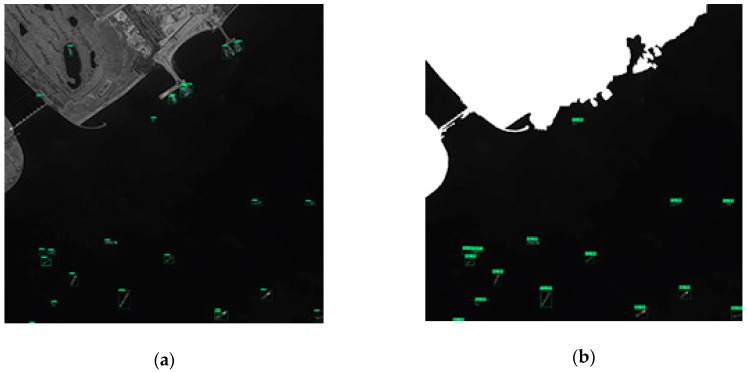
The detection results of the partial region of the GF-2(1) image. (**a**) Test results using network with a proportion of positive to negative samples of 1:1. (**b**) Test results using network with a proportion of positive to negative samples of 1:1 after FCN-based segmentation.

The first GF-2 image has obvious sea–land boundaries, higher resolution and small image size, and almost no ships on the coast, so that the detection performance of the image are all better than those of GF-1 (as shown in Table 6). The method incorporating negative sample training has only a small advantage in the detection performance. In the networks training with different positive and negative sample proportions, the network with the proportion of 1:2 has advantages in the detection performance. The detecting results of the partial region of the first panchromatic GF-2 image are shown in Figure 10. It can be seen that the sea–land segmentation method divides the coastal ship into terrestrial areas, which results in missed detection. Therefore, the method based on sea–land segmentation has a low recall.

(3) The second panchromatic GF-2 image with a spatial resolution of 0.81 m and size of 10,000 × 10,000.

**Figure 11:**
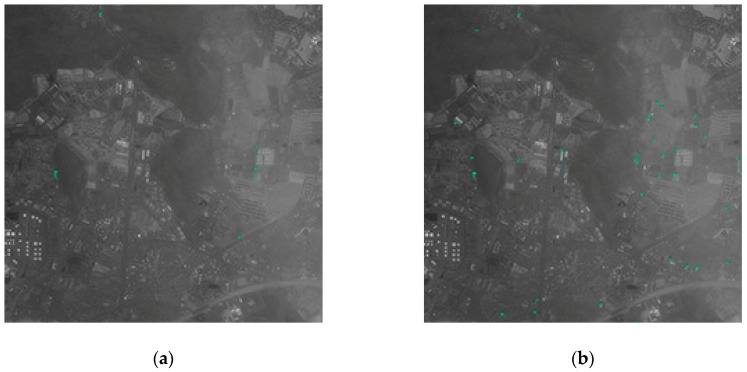
The detection results of the partial region of the GF-2(2) image. (**a**) Test results using network with a proportion of positive to negative samples of 1:1. (**b**) Test results using network with a proportion of positive to negative samples of 1:0.

The second GF-2 image is covered by large thin clouds, so that the sea-land boundary is blurred. Since the network training without negative samples lacks training of more ships’ resemblances on land, it will be judged as the ship target when encountering a misty object on the shore. Therefore, the precision of the network training without negative samples is significantly lower than other networks (as shown in Table 7). It can be seen from the third line of Figure 7 that the FCN-based method has a better segmentation performance around harbors. Since the sea–land segmentation method completely shields the blurred objects on land, the precision of method based on sea–land segmentation is higher. However, coastal ships are also missed because they are misclassified into land area, which causes low recall for the method based on sea–land segmentation. The detection result of the partial region of the second GF-2 image is shown in Figure 11.

(4) Two panchromatic JL-1 images with a spatial resolution of 0.72 m and size of 16,294 × 16,970.

**Figure 12:**
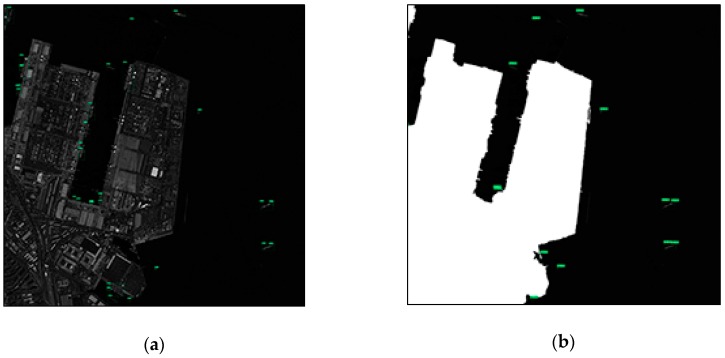
The detection results of the partial region of the JL-1(2) image. (**a**) Test results using network with a proportion of positive to negative samples of 1:1. (**b**) Test results using network with a proportion of positive to negative samples of 1:1 after FCN-based segmentation.

The two JL-1 images used in the experiment are images with complex environment around the port and high-resolution with a large proportion of the sea surface. It can be seen from the experimental results that since the land area is small; whether or not adding the negative sample to train the network has little effect on the experimental results (as shown in Table 8 and Table 9). Additionally, the network which adds less negative sample images can achieve better detection performance. However, due to the complicated environment around the port, too many shore ships lead to too many missed detections, which reduce its recall, using the sea–land segmentation method. The detecting results of the partial region of the two JL-1 images are shown in Figure 12.

## 5. Discussion

According to Figure 7 and Table 5, Table 6, Table 7, Table 8 and Table 9, we analyzed the experimental results in two parts: the first part discusses the overall comparison of proposed method and other methods for deep learning-based ship detection; the second part presents an analysis of suitable detection methods for different backgrounds.

### 5.1. Overall Comparison of Negative Sample Training and Others Based on R-CNN

As can be seen from the results section, although the deep learning-based sea–land segmentation method can avoid handcrafted extraction of features, it still has some inherent defects like traditional sea–land segmentation methods, such as the omission caused by dividing the landing ship and the large ship into terrestrial areas, the blurred coastline caused by clouds, and so on. Moreover, the preprocessing steps of sea–land segmentation increase the complexity of the algorithm. The non-negative sample method without sea–land segmentation preprocessing also degrades its detection performance due to false alarms caused by land area. This part quantitatively analyzes the experimental results with the R-CNN-based methods of negative sample training, non-negative sample training, and FCN-based sea–land segmentation for ship detection.

At the same time, a comparative experiment based on RPN with Zeiler and Fergus model (ZF) for ship detection, proposed by Kaikai et al. [24], is added. We implement this method with same hardware, software environment, and parameters required for training (Anchor scales of [0.25, 0.5, 1.0, 2.0], IoU of 0.5), and use the migration learning method to train sufficient on our dataset. After that, we tested the five large images using this method.

**Figure 13:**
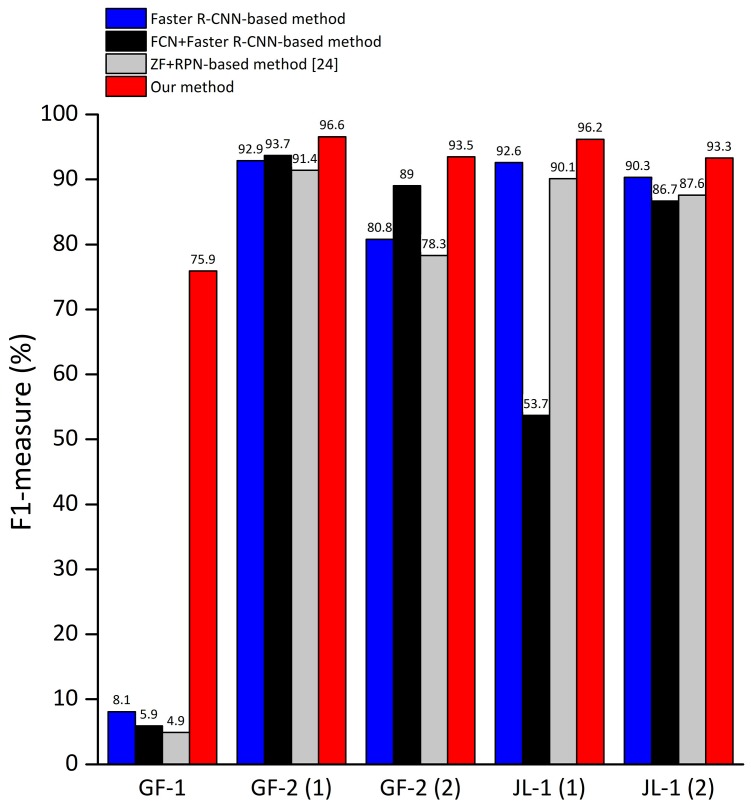
The F1-measure of the proposed method compared with other methods for deep learning-based ship detection.

Since F1-measure is an indicator which combines the recall and precision for comprehensively reflecting the overall detection performance. For the large images used in this paper, we calculated the F1-measure and drawn into a bar graph to more intuitively demonstrate the detection capabilities (as shown in Figure 13).

It can be seen from Figure 13 that the proposed method mostly can get better performance than others. Specifically, for images that contain large land area such as the GF-1 image, due to the proposed method reduces the false alarm caused by land and the sea–land segmentation method cannot get segmentation results with high accuracy in river region, the proposed method has a great improvement in detection performance and the absolute increment of F1-measure of our method can reach 70% compared to the ship detection method using FCN for sea–land segmentation. For images with simple coastlines and few coastal ships such as the GF-2 images, the F1-measure of our method is slightly better or equivalent to other methods. For images with complex harbors and many coastal ships, such as the JL-1 images, since methods based on sea–land segmentation are likely to cause missed detection of coastal ships, the absolute increment of F1-measure of our method can reach 42.5% compared to method based on sea–land segmentation. Moreover, the detection performance of the Faster R-CNN method using the Res101 network as the feature extraction network is better than the method using the ZF network as the feature extraction network proposed by Kaikai et al. [24]. From the experimental results of different images, it can be concluded that the proposed method has advantages in detection performance and robustness.

From the perspective of time consumption, the sea–land segmentation process takes a lot of time. The resulting images of sea–land segmentation obtained by preliminary segmentation methods need to be filled using mathematical morphology methods, so as to obtain the final complete sea–land segmentation results. Moreover, the method of sea–land segmentation preprocessing needs to divide the ship detection into two steps: preprocessing and detection. A larger image requires not only more computing time, but also more requirements for computer storage.

Therefore, the method incorporating negative sample training has advantages in terms of algorithm complexity, robustness, detection performance, and time consumption compared to the method of using sea–land segmentation preprocessing.

### 5.2. Comparison of Different Backgrounds

To different backgrounds for detection, the detection performance of different methods are different, so it is important to choose a suitable and robust detection method.

(1) Harbor

As can be seen from Figure 10 and Figure 12, the sea–land segmentation method is disadvantageous for ships in harbor. It can easily detect the docked ship as a land area which results in missed detection. The consequence of the miss of ship detection is very serious. Therefore, it is not recommended to adopt the sea–land segmentation method for the images containing complicated harbor.

(2) Land

According to Figure 9, in the case of a large area land of nonharbor, quantity false alarms will occur if the network training without a large number of negative samples. From Table 5, the GF-1 image that contains the large area nonharbor land shows that the method incorporating negative sample training can greatly improve the detection precision.

(3) Sea surface

For the sea surface background, the above methods can all achieve good performance.

(4) Rivers

Due to the complicated situation near the river, the boundaries of sea–land segmentation are not obvious and the sea–land segmentation performance is not very good. The rivers usually pass through a large number of land area, therefore, for the background of river regions, the method incorporating negative sample training is till recommended.

(5) Clouds

In the case of thin cloud cover, such as Figure 11, due to the unclear outline of the object in the land area, there will be some false alarms in the land area without sea–land segmentation. Since the sea–land segmentation method completely shields the blurred objects on land, the precision of the method based on sea–land segmentation is higher. But the sea–land segmentation method is still not good for handling the question of coastal ship. Due to the thin cloud, the coastline becomes more blurred. It increases the difficulty of sea–land segmentation and makes the performance of sea–land segmentation worse. At this time, if there is a missed detection on the coastal ship, the recall rate will be lower using sea–land segmentation. Therefore, in the case where the image is covered by thin cloud, these methods should be selectively chosen according to specific condition.

## 6. Conclusions

To avoid false alarms on land for deep learning-based ship detection, this paper proposes a ship detection method based on Faster R-CNN for large-scale optical images without sea–land segmentation. The proposed method adds a large number of images containing only land area without any ship targets as negative samples to avoid the false alarm on land, which is different from the selection of negative samples by targeted way in other detection methods. We designed experiments using the GF-1, GF-2, and JL-1 satellite images as testing images with different ship detection conditions to evaluate the effectiveness of the proposed strategy and compare with method based on sea–land segmentation. Based on the analysis of experimental results, the following conclusions can be obtained.

(1) The method incorporating negative sample training can almost achieve no false alarms in terrestrial areas without increasing labor, and is superior in detection performance, algorithm complexity, robustness, and time consumption than that of using sea–land segmentation, and can also adapt to various ship detection environments.
(2) Taking images in the paper as an example, for images that contain large land area such as the GF-1 image, the absolute increment of F1-measure in our method can reach 70%. For images with simple coastlines and few coastal ships such as the GF-2 images, the F1-measure of our method is slightly better or equivalent to other methods. And, for images with complex harbors and many coastal ships such as the JL-1 images, the absolute increment in F1-measure of our method can reach 42.5% compared to method based on sea–land segmentation.
(3) The proportion of sea to land of image area has an impact on the network detection performance. It is wise to choose a similar proportion of negative sample images based on the proportion of land area.

However, the test performance of the method incorporating negative sample training still have room for improvement. The possible future research directions are as follows.

(1) For smaller ships with wakes that have a faster moving speed on the sea surface, the network has a high rate of omission ratio. This question should be considered in the future works.
(2) As the region proposal of positive direction is not very effective in the task of detecting densely arranged ships, we will consider ship detection with rotation region proposal in the future.
(3) Design deep learning networks that are more suitable for small target detection to improve the precision of object detection.

## Figures and Tables

**Table 1:** Information of small image dataset.

The Number of Negative Sample Images	The Number of Positive Sample Images	The Number of Ships	Size	Satellite and Spatial Resolution	The Proportion of Training, Evaluation, and Test Images
2303	1282	5160	800 × 800	GF-2 0.81 m GF-1 2 m	16:4:5

**Table 2:** Comparison of ship detection results.

Deep Network for Ship Detection	mAP	Average Test Time Spent for a 800 × 800 Image (s)
SSD	0.482	0.0596
FPN	0.654	0.1605
Faster R-CNN	0.696	0.1848

**Table 3:** Information of networks training with different proportions of positive and negative sample images.

The Proportion of Positive and Negative Sample Images	The Number of Negative Sample Images	Training Steps	Training Time Spent (h)
1:0	0	49,000	21
2:1	645	75,000	32
1:1	1282	53,000	22.5
1:2	2303	140,000	61

**Table 4:** Information of five large images.

Image Name	Image Size (Pixels)	Satellite	Spatial Resolution	Number of Ships	Image Position
GF-1	21,227 × 21,227	GF-1	2 m	55	Hamburg, Germany (N53.50 E9.80)
GF-2(1)	10,000 × 10,000	GF-2	0.81 m	133	Straits, Singapore (N1.20 E103.80)
GF-2(2)	10,000 × 10,000	GF-2	0.81 m	149	Laem Chabang, Thailand (N13.10 E100.80)
JL-1(1)	16,294 × 16,970	JL-1	0.72 m	287	Tokyo Bay, Japan (N35.41 E139.63)
JL-1(2)	16,294 × 14,294	JL-1	0.72 m	208	Tokyo Bay, Japan (N35.33 E139.60)

**Table 5:** Results and time spent of detection of the GF-1 image.

The Proportion	Non-Sea-Land Segmentation				FCN-Based Segmentation			
	Time (s)	Detection Result			Time (s)	Detection Result		
		R	P	F		R	P	F
1:0	1497	0.385	0.045	0.081	117,683	0.034	0.216	0.059
2:1	**1463**	0.745	0.757	0.751	118,975	0.195	0.311	0.240
1:1	1470	**0.755**	0.764	**0.759**	116,984	0.103	0.461	0.168
1:2	1472	0.707	**0.788**	0.745	118,559	0.103	0.461	0.168

**Table 6:** Results and time spent of detection of the first GF-2 image.

The Proportion	Non-Sea–Land Segmentation				FCN-Based Segmentation			
	Time (s)	Detection Result			Time (s)	Detection Result		
		R	P	F		R	P	F
1:0	556	0.970	0.891	0.929	12,596	0.933	0.942	0.937
2:1	550	0.972	0.928	0.949	12,258	0.937	0.942	0.939
1:1	527	0.986	0.892	0.937	11,769	0.893	0.935	0.914
1:2	**509**	**0.992**	**0.942**	**0.966**	12,561	0.830	0.940	0.882

**Table 7:** Results and time spent for detection of the second GF-2 image.

The Proportion	Non-Sea–Land Segmentation				FCN-Based Segmentation			
	Time (s)	Detection Result			Time (s)	Detection Result		
		R	P	F		R	P	F
1:0	547	0.984	0.685	0.808	2756	0.813	0.982	0.890
2:1	542	**0.987**	0.871	0.925	2759	0.887	0.973	0.928
1:1	512	0.979	0.894	**0.935**	2416	0.867	**0.985**	0.922
1:2	**494**	0.972	0.884	0.926	2433	0.891	0.963	0.926

**Table 8:** Results and time spent of detection of the first JL-1 image.

The Proportion	Non-Sea–Land Segmentation				FCN-Based Segmentation			
	Time (s)	Detection Result			Time (s)	Detection Result		
		R	P	F		R	P	F
1:0	1740	0.970	0.885	0.926	35,245	0.374	**0.953**	0.537
2:1	1619	**0.987**	0.938	**0.962**	35,855	0.354	0.924	0.512
1:1	1580	0.980	0.930	0.954	34,216	0.325	0.945	0.484
1:2	**1576**	0.976	0.909	0.941	37,758	0.311	0.928	0.466

**Table 9:** Results and time spent of detection of the second JL-1 image.

The Proportion	Non-Sea–Land Segmentation				FCN-Based Segmentation			
	Time (s)	Detection Result			Time (s)	Detection Result		
		R	P	F		R	P	F
1:0	1742	0.974	0.841	0.903	36,418	0.817	0.924	0.867
2:1	1625	**0.974**	0.895	**0.933**	38,524	0.824	**0.945**	0.880
1:1	1595	0.961	0.864	0.910	37,581	0.807	0.922	0.861
1:2	**1585**	0.971	0.821	0.890	35,526	0.804	0.928	0.862
